# Chronic Lymphocytic Leukemia of the Aortic Valve: A Case Report

**DOI:** 10.7759/cureus.12517

**Published:** 2021-01-06

**Authors:** Marie-Louise Posch, Sean-Patrick A Prince, Kevin Thompson, Fagunkumar Modi, Jeffrey Jordan

**Affiliations:** 1 Internal Medicine, Citrus Memorial Hospital, Inverness, USA; 2 Pulmonology / Critical Care, Citrus Memorial Hospital, Inverness, USA

**Keywords:** cll, valve, leukemia, aortic, lymphocytic

## Abstract

Chronic lymphocytic leukemia (CLL) is characterized by the chronic accumulation of mature B-cell lymphocytes in the bone marrow. CLL accounts for approximately one-quarter of new leukemia cases each year and is the most common leukemia in Western countries. Most notably, this leukemia involves the lymph nodes, spleen, and liver, whereas non-lymphoid tissue is seldom associated with CLL infiltration. A large percentage of patients are asymptomatic at presentation; however, for those who are symptomatic, lymphadenopathy is the most common presenting complaint. This is the case of a 75-year-old Caucasian male with CLL on ibrutinib who presented with chest pressure and worsening shortness of breath. The patient underwent cardiac catheterization, which revealed demonstrable aortic stenosis. His aortic valve was subsequently replaced, and tissue was sent for histochemical analysis. Stains were positive for CD20, BCL2, CD5, and CD23, compatible with the CLL of the valve. To be able to investigate those with a known leukemic disease in patients with valvular disease would be beneficial to clinicians as CLL can present in atypical locations.

## Introduction

Chronic lymphocytic leukemia (CLL) is the most frequently diagnosed leukemia in the U.S. [1]. It is characterized by chronic accumulation of mature B-cell lymphocytes in bone marrow with classic findings such as lymphadenopathy, hepatomegaly, or weight loss. Patients typically present asymptomatically when a blood count demonstrates a lymphocytosis at the time of diagnosis [2]. CLL most commonly involves the lymph nodes, the spleen, and the liver, whereas non-lymphoid tissue is rarely associated with CLL infiltration [3, 4]. Here we discuss an unusual case of CLL of the aortic valve in a 75-year-old male patient.

## Case presentation

**Figure 1:**
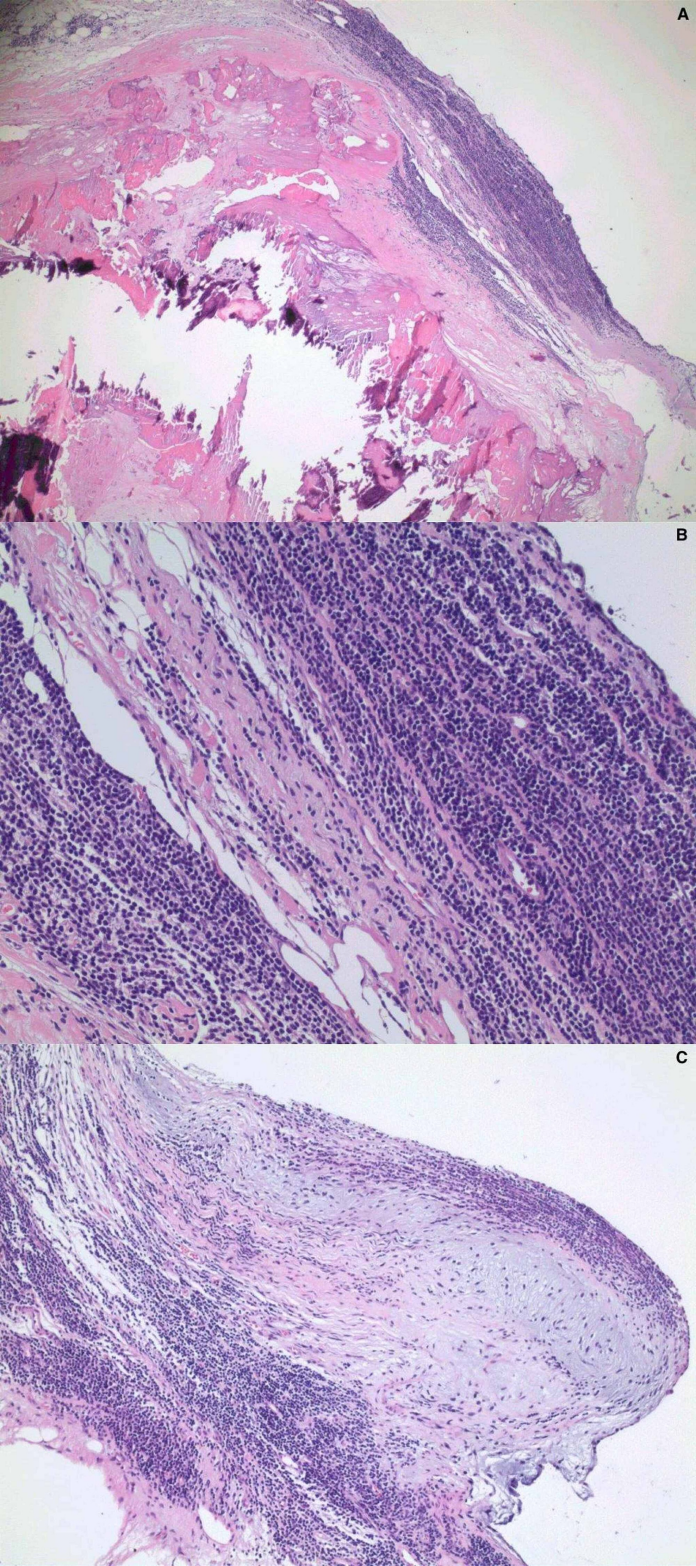
Aortic valve tissue featuring monotonous small lymphocytes

We report the case of a 75-year-old Caucasian male who was diagnosed with CLL in 2018. Past medical history was significant for diabetes, hypertension, coronary artery disease status post stents, moderate aortic stenosis, and atrial fibrillation. Two years later, the patient was complaining of chest heaviness with worsening exertional dyspnea and orthopnea. Cardiac catheterization was performed, which demonstrated diffuse re-stenosis of his stents and notable aortic stenosis. The patient underwent two-vessel coronary bypass surgery and aortic valve replacement. Prior to valve replacement, the patient’s oncologist started him on ibrutinib 420 mg daily due to worsening of his anemia and leukocytosis with significant adenopathy. In the setting of his CLL, the decision was made to send his atrial valve tissue for the histochemical examination, which revealed stains positive for CD20, BCL2, CD5, and CD23, consistent with CLL of the valve (Figure 1). The patient was continued on chemotherapy and followed up with his primary oncologist.

## Discussion

CLL accounts for about one-quarter of new leukemia cases each year and is the most common leukemia in Western countries [4]. It is characterized by clonal proliferation of CD5+, CD23+ B cells in the blood, bone marrow, and lymphoid tissue. It is estimated that about 25-50% of patients are asymptomatic at presentation. For symptomatic patients, lymphadenopathy is the usual complaint, followed by splenomegaly, hepatomegaly, pallor, or petechia [5]. Lymphadenopathy represents the spread of malignant lymphocytes from the bone marrow to the most common extramedullary involvement sites. As it spreads hematogenously, its growth can be somewhat predicted, as seen in other leukemia/lymphomas. As CLL progresses, the liver, lungs, skin, central nervous system, gastrointestinal tract, lungs, heart, and bone can become involved. Ratterman et al., in the systematic analysis of cases reported between 1975 and 2012, states that the central nervous system (CNS) and skin were the most commonly identified sites of spread, with some evidence that prognosis could be related to the location of involvement [6]. Less commonly, the involvement of the prostate, gallbladder, pituitary, thyroid, orbit, nasal mucosa has been reported [5, 7]. Rarely, endocardial deposits can be seen with intracavitary growth and even rarer tumor infiltration of heart valves [8]. Although metastatic disease to the heart is believed to be underestimated, it has been reported to occur between 0.7% and 3.5% in the general population [9]. Post mortem studies have shown cardiac metastases in up to one-fourth of patients who died of malignancy, and leukemic infiltrates of the heart in a third of patients who die of leukemia [8].

We identified two reported cases of CLL manifesting in a cardiac valve [10, 11]. One case involved a 77-year-old male who presented with worsening chest pain for eight weeks. Imaging revealed severe aortic stenosis, for which he underwent aortic valve replacement. Two of the aortic cusps were covered with multiple hemorrhagic nodules. Histological analysis of the valve demonstrated dense infiltration of monotonous small lymphocytes, and stains were positive for CD20, PAX25, CD5, and CD23 [4]. The second reported case gave an account of a 48-year-old black male with a history of severe mitral valve regurgitation and a three-year history of diagnosed CLL. He presented due to worsening dyspnea and underwent surgical mitral valve replacement. The patient’s mitral valve was sent for pathology and was found to have fibrosis and heavy lymphocyte infiltration mixed with fibroblast and histiocytes. Although stains were not done, the patient was found to have lymphocytic infiltration in several organs, most likely due to his CLL progression [10].

All of the CLL cases referenced in this report involved patients who presented with clinical heart failure in the setting of pronounced valvular disease. Our patient had symptomatic aortic stenosis, for which he underwent valve replacement and had confirmed lymphocytic infiltrates of his valvular tissue. This further raised the question of whether a diseased heart valve is more predisposed to leukemic infiltrates and warrants further investigation. It also stressed the importance of high clinical suspicion in patients with known diseased heart valves with leukemia as infiltrates might spread hematogenously to the aforementioned locations.

## Conclusions

Physicians should be aware of uncommon presentations and sites of involvement that are possible in CLL. As CLL can present in unusual sites, it is worth investigating those with valvular disease who concomitantly have a leukemic disease. It also postulates whether those with known leukemia who do not have diseased heart valves develop leukemic infiltrates in that area.

## References

[1] Siegel RL, Miller KD, Jemal A (2020). Cancer statistics, 2020. CA Cancer J Clin.

[2] Hallek M, Cheson BD, Catovsky D (2018). iwCLL guidelines for diagnosis, indications for treatment, response assessment, and supportive management of CLL. Blood.

[3] Rai KR, Sawitsky A, Cronkite EP, Chanana AD, Levy RN, Pasternack BS (1975). Pasternack BS Clinical staging of chronic lymphocytic leukemia. Blood.

[4] Zhang S, Kipps TJ (2014). The pathogenesis of chronic lymphocytic leukemia. Annu Rev Pathol.

[5] Atwal D, Raval M, Firwana B, Ramos J, Sasapu A (2017). An unusual presentation of chronic lymphocytic leukemia. Avicenna J Med.

[6] Ratterman M, Kruczek K, Sulo S, Shanafelt TD, Kay NE, Nabhan C (2014). Extramedullary chronic lymphocytic leukemia: systematic analysis of cases reported between 1975 and 2012. Leuk Res.

[7] Rao V, Watkins R, Kaleem A, Cooke J, Wedgwood K (2011). Leukaemic infiltration of gall bladder - unusual presentation of occult chronic lymphocytic leukaemia. J Surg Case Rep.

[8] Reynen K, Köckeritz U, Strasser RH (2004). Metastases to the heart. Ann Oncol.

[9] Maleszewski JJ, Bois MC, Bois JP (2018). Neoplasia and the heart: pathological review of effects with clinical and radiological correlation. J Am Coll Cardiol.

[10] Meltzer V, Korompai FL, Mathur VS, Guinn GA (1975). Surgical treatment of leukemic involvement of the mitral valve. Chest.

[11] Chisté M, Vrotsos E, Zamora C, Martinez A (2013). Chronic lymphocytic leukemia/small lymphocytic lymphoma involving the aortic valve. Ann Diagn Pathol.

